# Cardioprotective Effects and Possible Mechanisms of Luteolin for Myocardial Ischemia-Reperfusion Injury: A Systematic Review and Meta-Analysis of Preclinical Evidence

**DOI:** 10.3389/fcvm.2022.685998

**Published:** 2022-04-25

**Authors:** Qinyuan Pan, Yang Liu, Wenrui Ma, Rongsheng Kan, Hong Zhu, Dongye Li

**Affiliations:** ^1^Institute of Cardiovascular Disease Research, Xuzhou Medical University, Xuzhou, China; ^2^Department of Cardiology, The Affiliated Hospital of Xuzhou Medical University, Xuzhou, China

**Keywords:** luteolin, myocardial ischemia-reperfusion injury, apoptosis, oxidation, inflammation

## Abstract

**Background:**

At present, effective clinical therapies for myocardial ischemia-reperfusion injury (MIRI) are lacking. We investigated if luteolin conferred cardioprotective effects against MIRI and elucidated the potential underlying mechanisms.

**Method:**

Four databases were searched for preclinical studies of luteolin for the treatment of MIRI. The primary outcomes were myocardial infarct size (IS) and intracardiac hemodynamics. The second outcomes were representative indicators of apoptosis, oxidative stress, and inflammatory. The Stata and RevMan software packages were utilized for data analysis.

**Results:**

Luteolin administration was confirmed to reduce IS and ameliorate hemodynamics as compared to the control groups (*p* < 0.01). IS had decreased by 2.50%, 2.14%, 2.54% in three subgroups. Amelioration of hemodynamics was apparent in two different myocardial infarct models (model of left anterior descending branch ligation and model of global heart ischemia), as left ventricular systolic pressure improved by 21.62 and 35.40 mmHg respectively, left ventricular end-diastolic pressure decreased by 7.79 and 4.73 mmHg respectively, maximum rate of left ventricular pressure rise increased by 737.48 and 750.47 mmHg/s respectively, and maximum rate of left ventricular pressure decrease increased by 605.66 and 790.64 mmHg/s respectively. Apoptosis of cardiomyocytes also significantly decreased, as indicated by thelevels of MDA, an oxidative stress product, and expression of the inflammatory factor TNF-α (*p* < 0.001).

**Conclusion:**

Pooling of the data demonstrated that luteolin exerts cardioprotective effects against MIRI through different signaling pathways. As possible mechanisms, luteolin exerts anti-apoptosis, anti-oxidation, and anti-inflammation effects against MIRI.

## Introduction

Myocardial infarction is an acute heart condition characterized by decreased or complete cessation of blood flow to a portion of the myocardium, resulting in an imbalance between the supply and demand of oxygen to the myocardium and subsequent death of myocardiocytes (1, 2). Ultrastructural changes and mitochondrial abnormalities of cardiomyocytes are identified as early as 10 min after arterial occlusion (3, 4). However, cardiomyocyte necrosis can be detected hours later (5). The results of clinical studies have shown that appropriate and timely myocardial reperfusion therapy can effectively reduce ischemic injury (6, 7). Despite numerous benefits following recanalization of the coronary artery, postoperative mortality and morbidity remain significant (8). Moreover, the course of myocardial reperfusion itself can exacerbate myocardial systolic and diastolic function and expand myocardial infarct size (IS)–a phenomenon known as myocardial ischemia-reperfusion injury (MIRI) (9). Experimental studies have suggested that ~50% of the final IS is due to MIRI-induced cell death (10). From the emergence of this phenomenon, massive experimental studies of cardioprotective strategies against MIRI have been conducted (11). However, turning the application of these sorts of laboratory discoveries into treatments to improve patient outcomes have encountered significant obstacles primarily because of the multiple multi-factorial mechanisms underlying MIRI-induced cardiomyocyte injury (12). Hence, there is a need to assess the cardioprotective effects of potential strategies and elucidate the underlying mechanisms.

Although the herbs used in Traditional Chinese Medicine offer massive untapped potential for use in modern medicine, the underlying mechanisms remain unclear (13). Growing evidence suggests that dietary intake of flavonoids can reduce the incidence of ischemic heart disease (14). Luteolin, a 3', 4', 5, 7 tetra hydroxyl flavonoid derived from various plant sources, including broccoli, green pepper, and even peanut hulls, possess anti-apoptosis, antioxidant, anti-inflammatory, anti-tumor, and metabolic adjustment properties (15–18). So far, a variety of mechanisms of luteolin against MIRI have been identified. To accelerate the translation of cardioprotective effects of luteolin to clinical research, the empirical evidence and possible mechanisms of luteolin are summarized in this report.

Research on luteolin has been limited to preclinical trials, as most findings have been obtained from animal studies. However, the use of animal models has inherent flaws. For example, animal models generally only imitate a specific disease and cannot be implemented in adult animals without the induction of some comorbidities. Also, animal models are insufficient to reproduce the complicated pathophysiology in older adults with additional risk factors of myocardial infarction due to intrinsic heterogeneity. In addition, the conclusions of animal experiments are generally obtained from relatively small independent samples. Nonetheless, although animal studies are still necessary prior to preclinical studies, a well-designed quantitative meta-analysis with appropriate inclusion criteria can provide convincing evidence while minimizing bias. Hence, the aim of this review article was to summarize current knowledge of the cardioprotective effects of luteolin for treatment of MIRI.

## Methods

### Search Strategy

**Figure 1:**
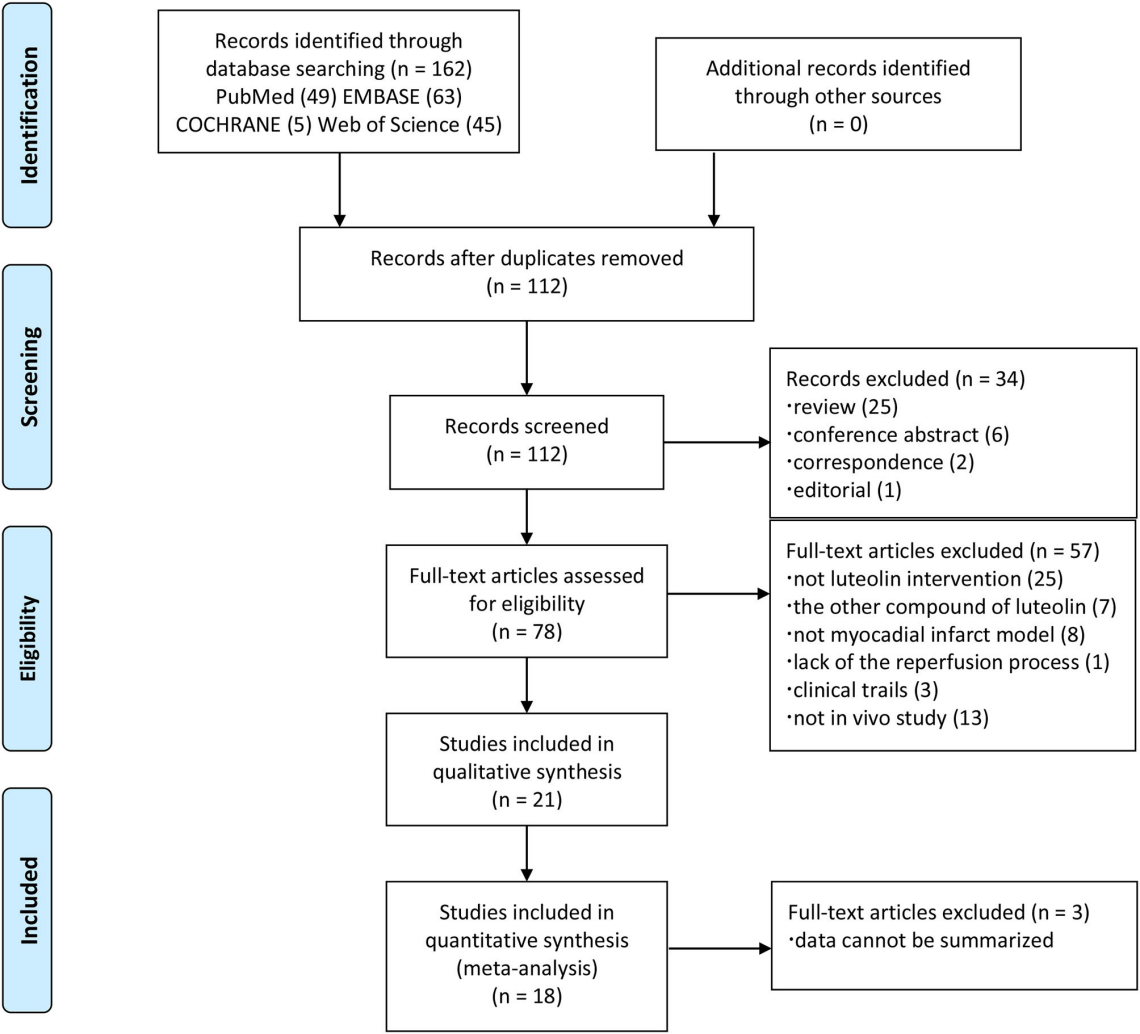
PRISMA flow chart of the search process.

Relevant articles published up to February 15, 2022 were retrieved from the PubMed, Embase, Cochrane Library, and Web of Sciencedatabases using the key words “luteolin” and “myocardial ischemia” without limitations to the year of publication, article type, or species. The PubMed database was searched with the use of the following retrieval statement: {[(Myocardial Ischemia) OR (Myocardial Ischemias) OR (Ischemias, Myocardial) OR (Ischemia, Myocardial) OR (Heart Disease, Ischemic) OR (Ischemic Heart Disease) OR (Heart Diseases, Ischemic) OR (Diseases, Ischemic Heart) OR (Disease, Ischemic Heart) OR (Ischemic Heart Diseases)] AND [(Luteolin) OR (3′,4′,5,7-Tetrahydroxy-Flavone) OR (3′,4′,5,7-Tetrahydroxyflavone) OR (Luteoline)]}. Quotations in eligible articles were also traced to minimize the possibility of omission as much as possible. The ID of PROSPERO is CRD42021226773. The search strategy and exclusion criteria are presented in Figure 1.

### Inclusion Criteria

Articles regarding preclinical studies on the Post-infarction cardioprotective effects of luteolin that met the following inclusion criteria were included in the meta-analysis: (1) use of an acute myocardial ischemia model induced by occlusion of the left anterior descending artery (LAD) or global Non-flow ischemia of the isolated heart; (2) the intervention group received pretreatment with luteolin monotherapy, while the MIRI group was administered normal saline, a vehicle, or no treatment with no restriction on the route of administration, time of pretreatment, or dosage; and (3) an experimental study with the use of an animal model with no restriction to the species, weight, or age, with the exception of sex. The primary indicators for evaluation were IS and hemodynamics. The hemodynamic indicators, as recorded with a pressure sensor, included: (1) left ventricular systolic pressure (LVSP); (2) left ventricular end-diastolic pressure (LVEDP); (3) maximum rate of left ventricular pressure rise (+dp/dt_max_); and (4) maximum rate of left ventricular pressure decrease (-dp/dt_max_). Secondary indicators, which included cardiomyocyte apoptotic rate, oxidative products, and inflammatory factors, reflected possible mechanisms of luteolin for treatment of MIRI.

### Exclusion Criteria

The exclusion criteria included the following: (1) no mention of the process for reperfusion of the impaired myocardium; (2) editorials, comments, conference abstracts, correspondences, reviews, and case reports; (3) pretreatment with other flavonoids or the other compound of luteolin; (4) *in vitro* studies; (5) pronounced intrinsic cardiac effect of anesthetics; and (6) clinical studies.

### Data Extraction

The data from the articles that met the inclusion criteria were included in a table with the following categories: (1) surname of the author and year of publication; (2) characteristics of the experimental animals of each study, which included sex, weight, and age; (3) establishment of an MIRI model and time of ischemia/reperfusion process; (4) staining and mode of identification of the infarcted myocardium; (5) method of treatment, including the administration route, dosage, and duration; (6) type of anesthetic; (7) outcome measures; and (8) related signaling pathways and corresponding inhibitors. The mean values and standard deviations of the control and intervention groups were extracted. If the results were indispensable but not explicitly shown, GetData Graph Digitizer software (http://getdata-graph-digitizer.com/) was used to assemble the data. When the luteolin dosages varied among the intervention groups, the data of which will be merged according the formula recommend by Cochrane Handbook (19). The formula is shown in Supplementary Figure 1. The indices detected by different methods in the same study were annotated as 1, 2, 3, etc. Data from the eligible articles were extracted by two independent authors and disputes were adjudicated by the corresponding author.

### Risk of Bias in Individual Studies

The checklist for animal data was rehashed as follows: (1) sample size estimation; (2) generation of random sequence; (3) no simulated myocardial ischemia; (4) blinding of outcome assessment; (5) appropriate animal model without comorbidities; (6) no noticeable intrinsic cardiac effect of anesthetics; (7) description of temperature control; (8) compliance with guidelines regarding the welfare of animals used in scientific testing and research; (9) publication after peer review; and (10) statement of a potential conflict of interest. If an entry qualified, the study received a score of 1 on a 10-point scale. Finally, the total score of each study was calculated. Any dispute was adjudicated by the corresponding author.

### Statistical Analysis

When there were differences in units or testing methods, or the presence of apparently exaggerated numbers, the extracted outcomes were converted to standardized mean difference (SMD) values with the 95% confidence interval (CI) to complete the summary statistics. In other cases, the weighted mean difference (WMD) and 95% CI were adopted. The sample size of an experimental study is usually smaller than of a clinical study. Therefore, Hedge's g rather than Cohen's d was implemented. Statistical heterogeneity between studies was calculated using the I squared (I2) statistic. Random effects were selected because of the small sample size and prevalent statistical heterogeneity in preclinical studies. To more accurately explore the effect of group size, prespecified subgroups were assessed by different methods. If heterogeneity was remarkable, subgroup analysis, sensitivity analysis, and/or meta-regression analysis were conducted. If the consistency of baseline data was poor, linear regression and multiple regression were utilized. If the same outcome index was reported by more than 10 articles, funnel plots and the Egger's test were used to assess publication bias. All quantitative outcomes were analyzed using Stata/SE version 12 (StataCorp LLC, College Station, TX, USA) and RevMan version 5.3 (The Cochrane Collaboration, London, England). A probability value of < 0.05 was considered statistically significant.

## Results

### Study Inclusion

In total, 162 articles were harvested in accordance with the search strategy and tracing of quotations. After the removal of 50 duplications, the remaining 112 studies were screened by browsing the article type. In addition, 25 reviews, six conference abstracts, two correspondences, and one editorial were also excluded as Non-original articles. Of the remaining 78 articles, 58 were also excluded: 25 because luteolin was not the primary focus or was mixed with other flavonoids, seven because of the other compound forms of luteolin (luteolin-7-O-glucoside and luteolin-7-beta-D-glucoside), eight due to the lack of a MIRI model, one that lacked the reperfusion method, three because of clinical trails and thirteen were not *in vivo* study. Finally, data were pooled from 21 articles.

### Study Characteristics and Baseline Data Analysis

All 21 articles (20–40) were published within the past 10 years. Sixteen studies (20–28, 30, 31, 35–38, 40) had healthy adult murids for comparison, and 5 studies (29, 32–34, 39) had comorbidities murids for comparison. Based on the construction method, two types of ischemia models were used in the included articles: ligation of LAD and Non-flow ischemia of the global heart. Nineteen studies (20, 22, 24–40) used rats as study subjects, while two articles (21, 23) used mice as study subjects. Eventually, eighteen articles (20, 23–28, 30–40) were included for quantitative analysis while two article (21, 23) was excluded by species and one article (29) was excluded to avoid the interference of comorbidity on the effect size. Eighty comparisons with 415 animals were tracked to describe amelioration of MIRI with luteolin, of which 263 were classified as the intervention group while 152 were classified as the control group. All of the animals were adult male rats, including Wistar rats with a weight range of 220–250 g, and Sprague Dawley rats with a weight range of 140–300 g. Anesthetics used for surgery that had no pronounced intrinsic cardiac effect included urethane, isoflurane, and pentobarbital sodium. Each experimental group was pretreated with luteolin via intravenous injection, intraperitoneal injection, gavage, or cardiac perfusion. The dosages differed via the administration route. There was also a dose-gradient design in some of the studies. The time of luteolin pretreatment spanned from dozens of minutes to 2 weeks before surgery. Nine articles (22, 24–26, 28, 30, 31, 35, 37) assessed IS by staining with 2,3,7-triphenyl tetrazolium-chloride (TTC) or Evans Blue/TTC. IS was assessed as the ratio of weight to area of IS. Areas stained by Evans blue were defined as an area not at risk, while the remaining area was defined as an area at risk (AAR), which was theoretically an area of IS without recanalization of the infarcted coronary artery. The AAR included the area of viable myocardium stained by TTC and the area of IS that negatively stained. The methods for calculation of the area of IS included IS/AAR, IS/whole heart, and IS/left ventricle (IS/LV). The duration of ischemia was 30 min. The duration of reperfusion was 24 h in five articles and 1–3 h, usually 2 h, in the others. Quantitative analysis in only one article reported the ejection fraction and fractional shortening. Consequently, intracardiac hemodynamic parameters for evaluation of heart function evaluation were included in 8 articles (20, 22, 27, 30, 31, 35–38). The fundamental characteristics of individual studies are shown in Table 1, and the mechanisms are listed in Table 2.

**Table 1:** Basic characteristics of the included studies.

**Study**	**Species, gender**	**Week old, weight**	**Model**	**Anesthetic**	**Method of treatment**			**Comorbidity**	**Performance**	**I/R time**	**Staining**	**Measurement of IS**
					**Luteolin**	**Duration**	**administration**					
Qin et al. (28)	SD rats, male	8–10 weeks, 240–260 g	MIRI	Pentobarbital sodium	40 mg/kg	3 d	po	no	ligation of LAD	4 h/12 h	NR	IS/ Whole Heart
Liu et al. (25)	SD rats, male	220–250 g	MIRI	Pentobarbital sodium	40 mg/kg	7 d	po	no	ligation of LAD	0.5 h/2 h	TTC	IS/ Whole Heart
Zhao et al. (38)	SD rats, male	8 weeks, 250–300 g	MIRI	Urethane	20/40 mg/kg	7 d	NR	no	ligation of LAD	0.5 h/2 h	NR	NR
Hu et al. (23)	C57BL/6j rats, male	Adult, 20–25 g	MIRI	Isoflurane	15 ug/kg	3 d	iv	no	ligation of LAD	0.5 h/24 h	EB/TTC	IS/AAR
Wei et al. (30)	SD rats, male	7–8 weeks, 200–250 g	MIRI	Pentobarbital sodium	5/10/20 mg/kg	15 min	ip	no	ligation of LAD	0.5 h/24 h	EB/TTC	IS/AAR
Du et al. (21)	C57BL/6j rats, male	NR, 20–25 g	MIRI	Pentobarbital sodium	15 ug/kg	3 d	iv	no	ligation of LAD	0.5 h/24 h	EB/TTC	IS/AAR
Zhang et al. (37)	SD rats, male	Adult. 220–250 g	MIRI	Isoflurane	40/80/160 mg/kg	7 d	po	no	ligation of LAD	0.5 h/24 h	TTC	IS/Whole Heart
Yu et al. (35)	SD rats, male	6–8 weeks, 250–300 g	MIRI	Urethane	10/40/70 mg/kg	5 d	po	no	ligation of LAD	0.5 h/1 h	TTC	IS/Whole Heart
Nai et al. (26)	SD rats, male	NR, 250–300 g	MIRI	Pentobarbital sodium	200 mg/kg	14 d	po	no	ligation of LAD	0.5 h/24 h	TTC	IS/LV
Sun et al. (29)	SD rats, male	Adult, 200–220 g	MIRI	Isoflurane	10 ug/kg	3 d	iv	DM	ligation of LAD	0.5 h/3 h	EB/TTC	IS/LV
Liao et al. (24)	SD rats, male	NR,250–300 g	MIRI	Urethane	10 ug/kg	15 min	iv	no	ligation of LAD	1 h/3 h	EB/TTC	IS/LV;IS/AAR
Zhou et al. (39)	SD rats, male	Adult,210–220 g	MIRI	Pentobarbital sodium	100 mg/kg	14 d	po	DM	global ischemia of heart	0.5 h/2 h	NR	NR
Xiao et al. (32)	SD rats, male	NR, 210–230 g	MIRI	Pentobarbital sodium	100 mg/kg	14 d	po	DM	global ischemia of heart	0.5 h/2 h	NR	NR
Yang et al. (34)	SD rats, male	NR, 140–180 g	MIRI	Pentobarbital sodium	100 mg/kg	14 d	po	hyperlipoidemia	global ischemia of heart	0.5 h/2 h	NR	NR
Zhu et al. (40)	Wistar rats, male	NR, 220–250 g	MIRI	Pentobarbital sodium	40 μM	30 min	perfusion	no	global ischemia of heart	0.5 h/2 h	NR	NR
Zhang et al. (36)	SD rats, male	NR,220–250 g	MIRI	Pentobarbital sodium	40 μM	20 min	perfusion	no	global ischemia of heart	0.5 h/2 h	NR	NR
Yang et al. (33)	SD rats, male	NR, 220–240 g	MIRI	Pentobarbital sodium	100 mg/kg	14 d	po	DM	global ischemia of heart	0.5 h/2 h	NR	NR
Bian et al. (20)	SD rats, male	NR,220–250 g	MIRI	Pentobarbital sodium	40 μM	30 min	perfusion	no	global ischemia of heart	0.5 h/2 h	NR	NR
Wu et al. (31)	Wistar rats, male	NR,220–250 g	MIRI	Pentobarbital sodium	40 μM	30 min	perfusion	no	global ischemia of heart	0.5 h/2 h	TTC	IS/LV
Qi et al. (27)	SD rats, male	NR,220–250 g	MIRI	Pentobarbital sodium	10 μg/ml	10 min	perfusion	no	global ischemia of heart	0.5 h/2 h	NR	NR
Fang et al. (22)	SD rats, male	NR,220–250 g	MIRI	Pentobarbital sodium	40 μM	30 min	perfusion	no	global ischemia of heart	0.5 h/2 h	TTC	IS/LV

*SD, Sprague—Dawley; NR, not report; MIRI, myocardial ischemia/reperfusion injury; Duration, the duration of luteolin pretreatment; μM, μmol/L; d, day; h, hour; min, minute; DM, diabetes mellitus; po, intragastric administration; ip, intraperitoneal injection; iv, intravenous injection; I/R, ischemia/reperfusion; LAD, left anterior descending branch; IS, infarct size; EB, Evans blue; TTC, 2,3,7-triphenytetrazolium-chloride; AAR, area at risk; LV, left ventricular*.

**Table 2:** Summary of mechanisms of luteolin for MIRI.

**References**	**Outcome measure**	**Intergroup differences**	**Mechanisms**	**Interventions of signal pathways**
Qin et al. (28)	1. Infarct size 2. Apoptotic index 3. Oxidative factor	1. *p* < 0.05 2. *p* < 0.05 3. *p* < 0.05	Wnt↑/β-catenin↑/ oxidative stress↓; apoptosis↓	NR
Liu et al. (25)	1. Infarct size 2. Apoptotic index 3. Inflammatory factor	1. *p* < 0.05 2. *p* < 0.05 3. *p* < 0.05	SHP-1↓STAT3↑ /inflammatory reactions and cell death↓	NR
Zhao et al. (38)	1. Hemodynamics 2. Cardiac enzyme 3. Inflammatory factor	1. *p* < 0.01 2. *p* < 0.01 3. *p* < 0.01	Siti1/NLRP3/NF-κB pathway↓	NR
Hu et al. (23)	1. Hemodynamics 2. Cardiac enzyme 3. Infarct size 4. Apoptotic index	1. *p* < 0.001 2. *p* < 0.001 3. *p* < 0.001 4. *p* < 0.001	Sp1↑/SERCA2a↑;apoptosis↓	Sp1 overexpression and Sp1 knockdown
Wei et al. (30)	1. Infarct size 2. Cardiac enzyme 3. Oxidative factor 4. Hemodynamics	1. *p* < 0.05 2. *p* < 0.05 3. *p* < 0.05 4. *p* < 0.05	PRXII↑/oxidative stress↓/ apoptosis↓	Conoidin A (a specific covalent inhibitor of PRXII)
Du et al. (21)	1. Infarct size 2. Cardiac enzyme 3. Hemodynamics 4. Apoptotic index	1. *p* < 0.001 2. *p* < 0.001 3. *p* < 0.001 4. *p* < 0.001	SERCA2a↑via its Sumoylation at Lysine 585	NR
Zhang et al. (37)	1. Infarct size 2. Hemodynamics 3. Cardiac enzyme 4. Inflammatory factor	1. *p* < 0.05 2. *p* < 0.01 3. *p* < 0.01 4. *p* < 0.01	TLR4/NF-kB/NLRP3 inflammasome pathway↓	NR
Yu et al. (35)	1. Hemodynamics 2. Infarct size 3. Cardiac enzyme 4. Oxidative factor 5. Apoptotic index	1. *p* < 0.01 2. *p* < 0.01 3. *p* < 0.01 4. *p* < 0.01 5. *p* < 0.05	ROS-activated MAPK pathway↓/apoptosis↓	SB203580 (p38 MAPK inhibitor) SP600125 (JNK MAPK inhibitor)
Nai et al. (26)	1. Infarct size 2. Cardiac enzyme 3. Apoptotic index	1. *p* < 0.01 2. *p* < 0.01 3. *p* < 0.01	PI3K/Akt signal pathway↑/SERCA2a↑	LY294002 (the Akt inhibitor)
Sun et al. (29)	1. Cardiac enzyme 2. Infarct size 3. Hemodynamics 4. Apoptotic index 5. Inflammatory factor	1. *p* < 0.05 2. *p* < 0.05 3. *p* < 0.05 4. *p* < 0.05 5. *p* < 0.05	FGFR2↑and LIF↑/ apoptosis↓; PI3K/Akt pathway↑/inflammation↓; apoptosis↓	Wortmannin (a specific PI3K inhibitor)
Zhou et al. (39)	1. Hemodynamics 2. Cardiac enzyme 3. Oxidative factor	1. *p* < 0.01 2. *p* < 0.01 3. *p* < 0.01	sestrin2-mediated removal of Keap1/Nrf2↑/oxidative stress↓	Leucine (the sestrin2 inhibitor) Brusatol (the Nrf2 inhibitor)
Liao et al. (24)	1. Cardiac enzyme 2. Infarct size 3. Oxidative factor	1. *p* < 0.05 2. *p* < 0.05 3. *p* < 0.05	reduction in iNOS production	NR
Xiao et al. (32)	1. Hemodynamics 2. Cardiac enzyme 3. Oxidative factor	1. *p* < 0.01 2. *p* < 0.01 3. *p* < 0.01	eNOS-mediated S-nitrosylation of Keap1↑/Nrf2↑/oxidative stress↓	L-NAME (the NOS inhibitor) Brusatol (the Nrf2 inhibitor)
Yang et al. (34)	1. Hemodynamics 2. Cardiac enzyme 3. Oxidative factor	1. *p* < 0.01 2. *p* < 0.01 3. *p* < 0.01	enhancing Akt/GSK3β/Fyn-mediated Nrf2 antioxidative function	LY294002 (the Akt inhibitor)
Zhu et al. (40)	1. Apoptotic index	1. *p* < 0.01	p38MAPK pathway↓/apoptosis↓;SERCA2a↑	SB203580 (the p38 MAPK inhibitor)
Zhang et al. (36)	1. Oxidative factor 2. Hemodynamics	1. *p* < 0.01 2. *p* < 0.05	ROS↓/P38MAPK↓/apoptosis↓; PI3K/AKT↑/oxidative injury↓	LY294002 (the Akt inhibitor)
Yang et al. (33)	1. Hemodynamics 2. Cardiac enzyme 3. Oxidative factor	1. *p* < 0.01 2. *p* < 0.01 3. *p* < 0.01	eNOS pathway↑/MnSOD↑ and mPTP opening↓	L-NAME (the NOS inhibitor)
Bian et al. (20)	1. Hemodynamics 2. Apoptotic index	1. *p* < 0.05 2. *p* < 0.05	miR-208b-3p↓/ Ets1↑/apoptosis↓	overexpression and knockdown of miR-208b-3p
Wu et al. (31)	1. Hemodynamics 2. Infarct size 3. Apoptotic index	1. *p* < 0.05 2. *p* < 0.05 3. *p* < 0.05	ERK1/2↑and JNK↓/ apoptosis↓; ERK1/2-PP1a signal pathway↑/SERCA2a↑	PD98059(ERK1/2 inhibitor) SP600125(JNK inhibitor)
Qi et al. (27)	1. Apoptotic index 2. Hemodynamics	1. *p* < 0.01 2. *p* < 0.01	apoptosis↓;necrosis↓	NR
Fang et al. (22)	1. Hemodynamics 2. Infarct size 3. Apoptotic index	1. *p* < 0.05 2. *p* < 0.01 3. *p* < 0.01	PI3K/AKT pathway↑/apoptosis↓	LY294002 (the Akt inhibitor)

*NR, no reported; NLRP3, NLR Family, Pyrin Domain-Containing 3 Protein; sp1, Specificity Protein 1 Transcription Factor; SERCA2a, Sarcoplasmic Reticulum Ca2+-ATPase; PRX II, Peroxiredoxin II; TLR4, Toll-Like Receptor 4; MAPK, Mitogen-Activated Protein Kinase Kinases; PI3K, phosphoinositide 3-kinase; JNK, C-Jun N terminal kinase; ERKs, extracellular signal-regulated kinases; FGFR2, fibroblast growth factor receptor 2; sestrin2, a highly conserved stress-inducible protein; LIF, leukemia inhibitory factor; iNOS, inducible nitric oxide synthase; eNOS, endothelial nitric oxide synthase; GSK3β, Glycogen Synthase Kinase 3 beta; Nrf2, nuclear factor erythroid 2-related factor 2; MPTP, mitochondrial membrane permeability transition pore*.

In the group of LAD ligation, baseline data (i.e., administrations, dosages, pretreatment timing, and reperfusion duration) were inconsistent. It has been reported that oral bioavailability of luteolin was 26 ± 6% while intravenous bioavailability was usually set at 100% (41). Although the method of administration was different, the included studies had explored the optimal dosage or quoted the dosage designed by others. Regression analysis analysis by administration, dosages, timing regimen of pretreatment or reperfusion duration had no impact on the effect size of IS and hemodynamics (Supplementary Table 1). The different Research Groups have explored their optimal experimental conditions for the best experimental results. Hence, the effect size did not be significantly affected by baseline data. In the model of global ischemia, the baseline data except for reperfusion duration was consistent. Regression analysis by reperfusion duration had no impact on the effect size of hemodynamics (Supplementary Table 1).

### Study Quality and Publication Bias

The lowest study quality score was 6 points on a 10-point scale, while the highest was 9 points. Of the 21 completed studies, 19.05% were assigned a quality score of 6 points, 19.05% a score of 7 points, 52.38% a score of 8 points, and 9.52% a score of 9 points. The majority of studies received relatively high scores. All of the included studies were published in peer-reviewed journals. Control of temperature and animal welfare were described in 21 studies. No study mentioned sample size estimation. The process of randomly assigning animals to each group was described in all 21 studies. Outcomes were assessed blindly in five studies (22, 25, 27, 29, 30). A total of 16 studies (20–28, 30, 31, 35–38, 40) included a control group comprised of healthy adult animals without complications. The administration of anesthetics had hardly any effect on heart function in 21 articles. All studies declared potential conflicts of interest. The methodological quality of individual studies is shown in Table 3. The Egger test (*P* > 0.05) (Supplementary Figure 2) and the funnel plot (Supplementary Figure 3) showed no significant publication bias for all indicators except LVEDP (*P* = 0.014). However, trim and filling method illustrates the better authenticity of LVEDP results (Supplementary Figure 3C). The value of LVEDP changes from −6.35 to −9.03 (95% CI = −11.20 to −6.86, *P* < 0.001) through trim and filling method. The new valuation does not cross the invalid line.

**Table 3:** Risk of bias of included studies according to CAMARADES checklist.

**References**	**(1)**	**(2)**	**(3)**	**(4)**	**(5)**	**(6)**	**(7)**	**(8)**	**(9)**	**(10)**	**Total**
Bian et al. (20)		✓			✓	✓	✓	✓	✓	✓	7
Du et al. (21)		✓	✓		✓	✓	✓	✓	✓	✓	8
Fang et al. (22)		✓		✓	✓	✓	✓	✓	✓	✓	8
Hu et al. (23)		✓	✓		✓	✓	✓	✓	✓	✓	8
Liao et al. (24)		✓	✓		✓	✓	✓	✓	✓	✓	8
Liu et al. (25)		✓	✓	✓	✓	✓	✓	✓	✓	✓	9
Nai et al. (26)		✓	✓		✓	✓	✓	✓	✓	✓	8
Qi et al. (27)		✓		✓	✓	✓	✓	✓	✓	✓	8
Qin et al. (28)		✓	✓		✓	✓	✓	✓	✓	✓	8
Sun et al. (29)		✓	✓	✓		✓	✓	✓	✓	✓	8
Wei et al. (30)		✓	✓	✓	✓	✓	✓	✓	✓	✓	9
Wu et al. (31)		✓			✓	✓	✓	✓	✓	✓	7
Xiao et al. (32)		✓				✓	✓	✓	✓	✓	6
Yang et al. (34)		✓				✓	✓	✓	✓	✓	6
Yang et al. (33)		✓				✓	✓	✓	✓	✓	6
Yu et al. (35)		✓	✓		✓	✓	✓	✓	✓	✓	8
Zhang et al. (37)		✓	✓		✓	✓	✓	✓	✓	✓	8
Zhang et al. (36)		✓			✓	✓	✓	✓	✓	✓	7
Zhao et al. (38)		✓	✓		✓	✓	✓	✓	✓	✓	8
Zhou et al. (39)		✓				✓	✓	✓	✓	✓	6
Zhu et al. (40)		✓			✓	✓	✓	✓	✓	✓	7

*Studies fulfilling the criteria of: (1) sample size estimation; (2) the generating of random sequence; (3) no simulated myocardial ischemia; (4) blinding of outcome assessment; (5) appropriate animal model without comorbidities; (6) no pronounced intrinsic cardiac effect of anesthetic; (7) description of temperature control; (8) comply with animal protection laws; (9) the paper is published after peer review; (10) stated potential conflict of interest*.

### Effectiveness

#### Myocardial IS

**Figure 2:**
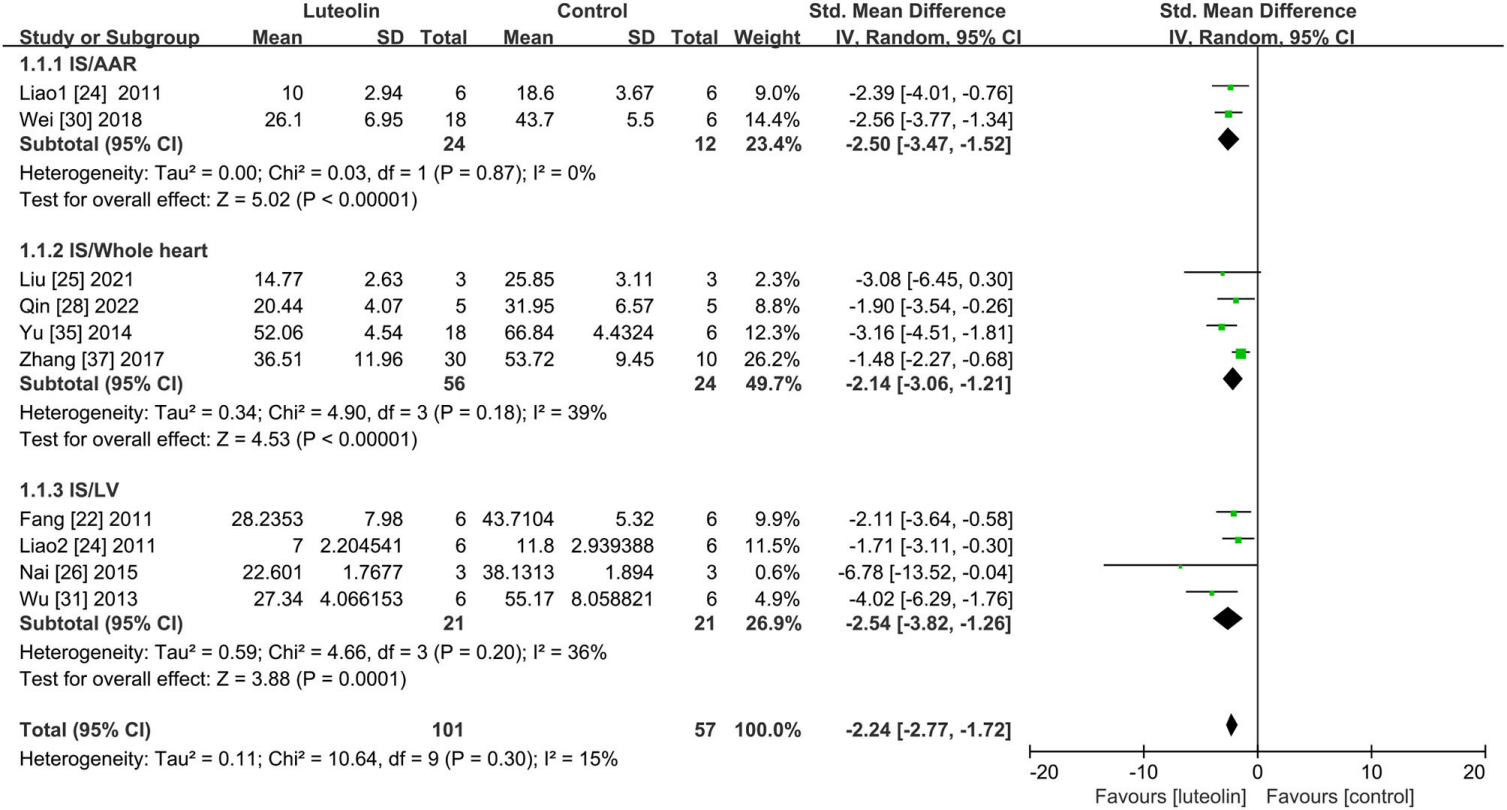
Forest plot showing changes in myocardial infarct size. IS, infarct size; AAR, area at risk; LV, left ventricular.

Subgroups were established based on IS measurements (Figure 2). Within the subgroups, SMD with the 95% CI was calculated to determine the coexistence of the area ratio and weight ratio. There were three subgroups of IS. Quantitative analysis of two studies (24, 30) showed that luteolin administration led to a decrease in the IS/AAR ratio as compared to that of the control group [Figure 2, SMD = −2.50, 95% CI = −3.47 to −1.52, *p* < 0.00001; heterogeneity: χ^2^ = 0.03, df = 2 (p = 0.87); I^2^ = 0%]. Quantitative analysis of four studies (25, 28, 35, 37) showed that the IS/whole heart ratio was significantly decreased as compared to that of the control group [Figure 2, SMD = 2.14, 95% CI = 3.06 to −2.14, *p* < 0.0001; heterogeneity: χ^2^ = 4.90, df = 3 (*p* = 0.18); I^2^ = 39%]. Quantitative analysis of four studies (22, 24, 26, 31) showed that luteolin administration reduced the IS/LV ratio as compared to that of the control group [Figure 2, SMD = −2.54, 95% CI = −3.82 to −1.26, *p* = 0.0001; heterogeneity: χ^2^ = 4.66 df = 3 (*p* = 0.20); I^2^ = 36%]. The subgroup analysis for the MIRI model was also conducted. In regard to the LAD ligation model, quantitative analysis of seven studies (24–26, 28, 30, 35, 37) with eight comparisons showed that luteolin administration reduced the IS as compared to that of the control group [Supplementary Figure 4, SMD = −2.14, 95% CI = −2.68 to −1.59, *p* < 0.00001; heterogeneity: χ^2^ = 7.94, df = 7 (*p* = 0.34); I^2^ = 12%]. In regard to the global ischemia model, quantitative analysis of two studies (22, 31) showed that luteolin administration reduced the IS as compared to that of the control group [Supplementary Figure 4, SMD = −2.87, 95% CI = −4.72 to −1.03, *p* = 0.002; heterogeneity: χ^2^ = 1.89, df = 1 (*p* = 0.17); I^2^ = 47%].

#### Hemodynamics

##### LVSP

**Figure 3:**
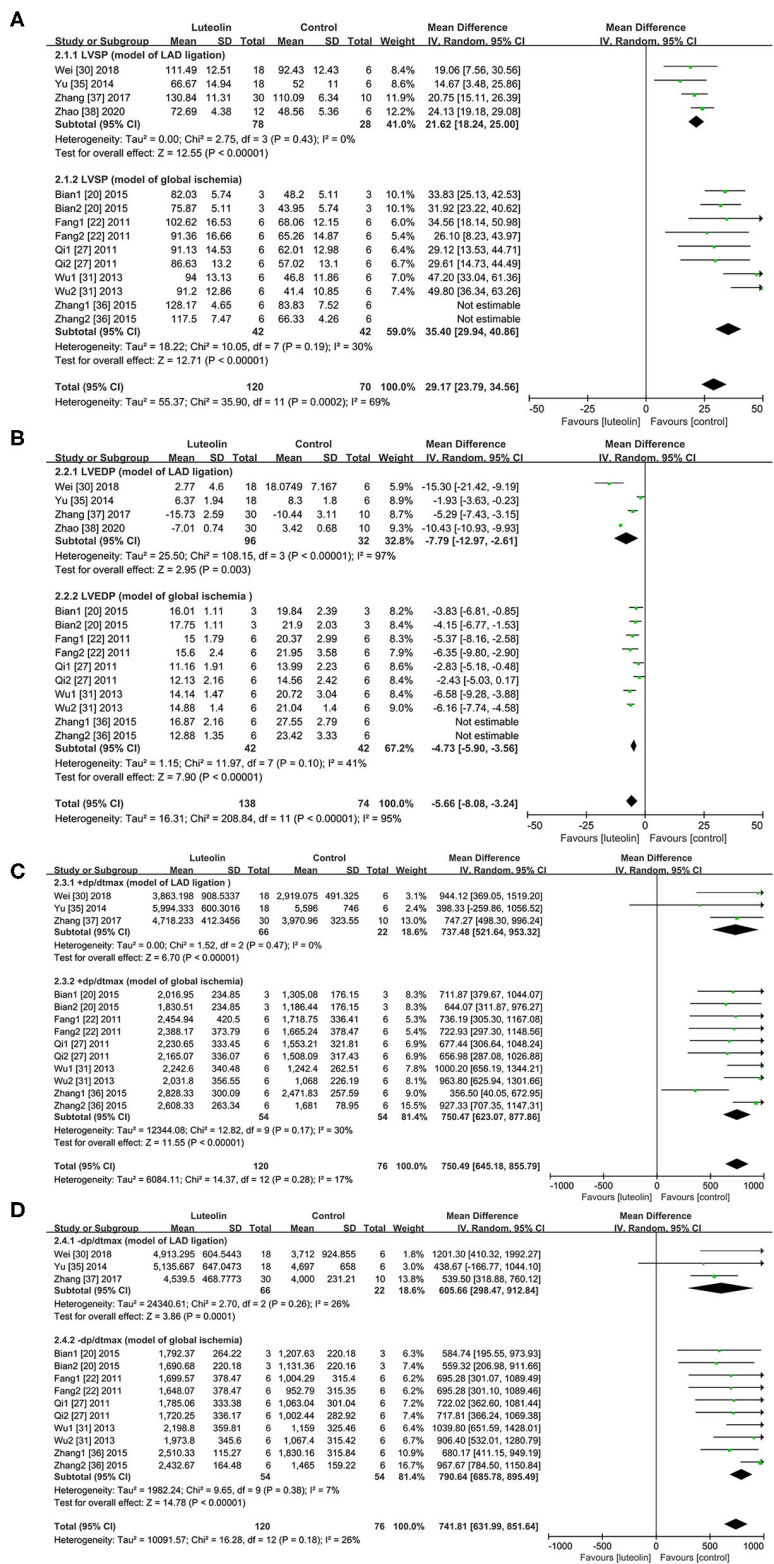
Forest plots for effect of luteolin on hemodynamics including **(A)** LVSP, **(B)** LVEDP, **(C)** +dp/dt_max_, **(D)** -dp/dt_max_. LVSP, left ventricular systolic pressure; LVEDP, left ventricular end-diastolic pressure; +dp/dt_max_, maximum rate of left ventricular pressure rise; -dp/dt_max_, maximum rate of left ventricular pressure decrease.

In regard to the LAD ligation model, quantitative analysis of four studies (30, 35, 37, 38) showed that LVSP was significantly improved in the luteolin group as compared to that of the control group [Figure 3A, WMD = 21.62, 95% CI = 18.24 to 25.00, *p* < 0.00001; heterogeneity: χ^2^ = 2.75, df = 3 (*p* = 0.43); I^2^ = 0%]. In regard to the global ischemia model, quantitative analysis of five studies (20, 22, 27, 31, 36) with 10 comparisons showed that LVSP was significantly improved in the intervention group as compared to that of the control group [Figure 3A, WMD = 38.82, 95% CI = 32.82 to 44.82, *p* < 0.00001; heterogeneity: χ^2^ = 26.14, df = 9 (*p* = 0.002); I^2^ = 66%]. Here, sensitivity analysis was performed to determine the source of heterogeneity (Supplementary Figure 5). After the removal of one study (36), quantitative analysis of four studies (20, 22, 27, 31) with eight comparisons showed that LVSP was significantly improved as compared to that of the control group [Figure 3A, WMD = 35.40, 95% CI = 29.94 to 40.86, *p* < 0.00001; heterogeneity: χ^2^ = 10.05, df = 7 (*p* = 0.19); I^2^ = 30%].

##### LVEDP

In regard to the LAD ligation model, Quantitative analysis of four studies (30, 35, 37, 38) showed that luteolin administration reduced LVEDP as compared to that of the control group [Figure 3B, WMD = −7.79, 95% CI = −12.97 to −2.61, *p* < 0.00001; heterogeneity: χ^2^ = 108.15, df = 3 (*p* < 0.00001); I^2^ = 97%]. Sensitivity analysis had no pronounced effect on the I^2^ values (Supplementary Figure 6).

In regard to the global ischemia model, three articles (32–34) that lacked a control group at the corresponding time point during reperfusion of the isolated heart were excluded from analysis. Thus, quantitative analysis of five studies (20, 22, 27, 31, 36) with 10 comparisons showed that luteolin administration significantly reduced LVEDP as compared to that of the control group [WMD = −5.85, 95% CI = −7.54 to −4.16, *p* < 0.00001; heterogeneity: χ^2^ = 38.43, df = 9 (*p* < 0.01); I^2^ = 77%]. After removal of one study (36) by sensitivity analysis (Supplementary Figure 7), the heterogeneity was decreased, indicating a meaningful change [Figure 3B, WMD = −4.73, 95% CI = −5.90 to −3.56, *p* < 0.01; heterogeneity: χ^2^ = 11.97, df = 7 (*p* = 0.10); I^2^ = 41%].

##### +dp/dt_max_

In regard to the LAD ligation model, quantitative analysis of three (30, 35, 37) studiesshowed that luteolin administration increased +dp/dt_max_ as compared to that of the control group [Figure 3C, WMD = 737.48, 95% CI = 521.64 to 953.32, *p* < 0.00001; heterogeneity: χ^2^ = 1.52, df = 2 (*p* = 0.47); I^2^ = 0%]. In regard to the global ischemia model, quantitative analysis of five studies (20, 22, 27, 31, 36) with 10 comparisons showed that luteolin administration increased +dp/dt_max_ as compared to that of the control group [Figure 3C, WMD = 750.47, 95% CI = 623.09 to 877.86, *p* < 0.01; heterogeneity: χ^2^ = 12.82, df = 9 (*p* = 0.17); I^2^ = 30%].

##### -dp/dt_max_

In regard to the LAD ligation model, quantitative analysis of three studies (30, 35, 37) showed that luteolin administration significantly increased -dp/dt_max_ as compared to that of the control group [Figure 3D, WMD = 605.66, 95% CI = 298.47 to 912.84, *p* = 0.0001; heterogeneity: χ^2^ = 2.70, df = 2 (*p* = 0.26); I^2^ = 26%].

In regard to the global ischemia model, quantitative analysis of five studies (20, 22, 27, 31, 36) with 10 comparisons showed that luteolin administration significantly increased -dp/dt_max_ as compared to that of the control group [Figure 3D, WMD = 790.64, 95% CI = 685.78 to 895.49, *p* < 0.01; heterogeneity: χ^2^ = 9.65, df = 9 (*p* = 0.38); I^2^ = 7%].

#### Cardioprotective Mechanisms of Luteolin

##### Anti-apoptosis

**Figure 4:**
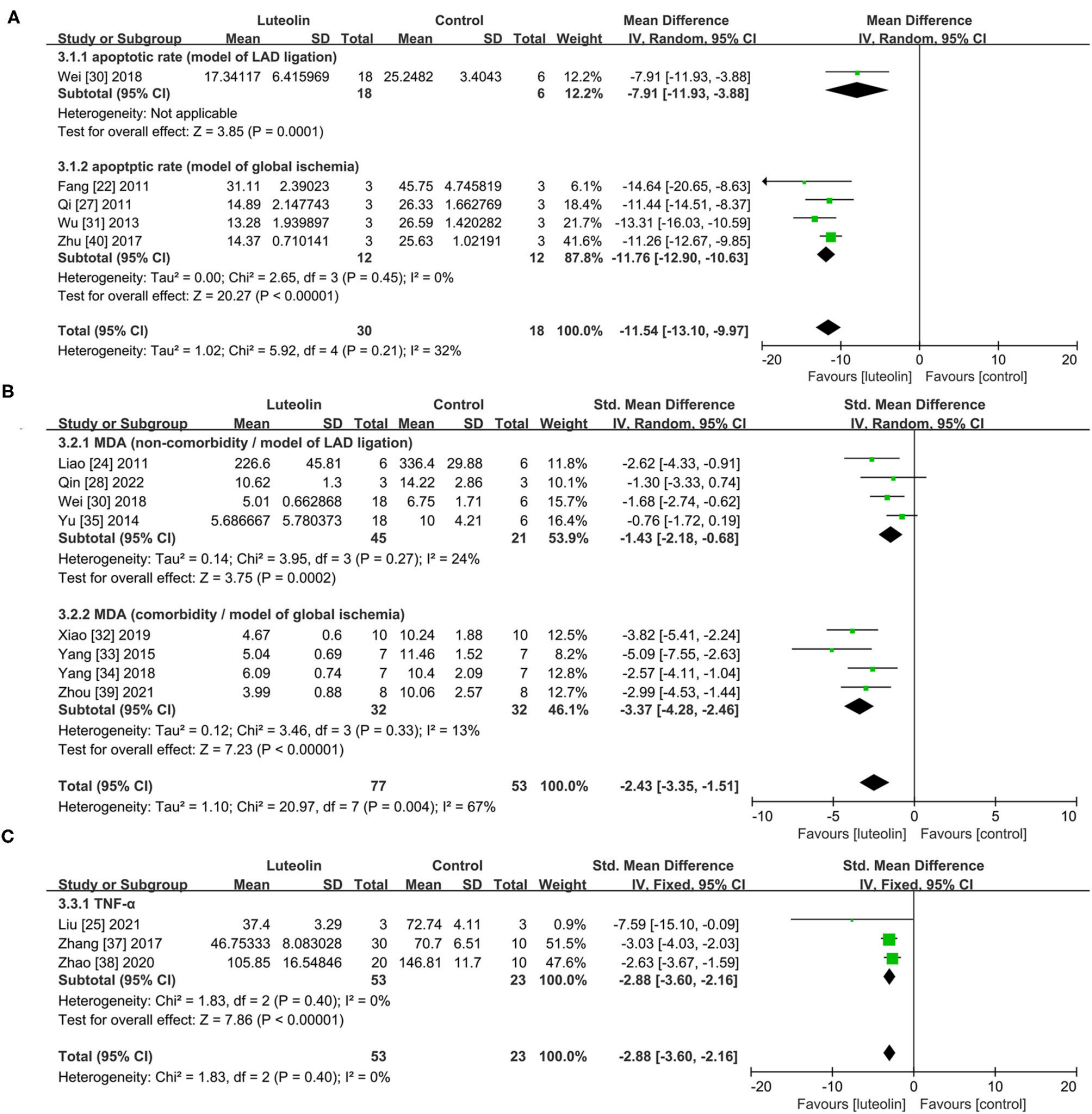
Forest plots for **(A)** apoptotic rate, **(B)** MDA, **(C)** TNF-α. LAD, left anterior descending branch; MDA, malondialdehyde; TNF-α, tumor necrosis factor alpha.

Only one study (30) included in the group of LAD ligation model. In regard to the global ischemia model, quantitative analysis of four studies (22, 27, 31, 40) showed that luteolin administration significantly decreased the proportion of apoptotic cells (TUNEL-positive cells) in the intervention group as compared to that of the control group [Figure 4A, WMD = −11.76, 95% CI = −12.90 to −10.63, *p* < 0.00001; heterogeneity: χ^2^ = 2.65, df = 3 (*p* = 0.45); I^2^ = 0%].

##### Anti-oxidation

Eight studies (24, 27, 30, 32–35, 39) showed that luteolin administration was significant for decreasing MAD level compared with the control group [Figure 4B, SMD = −2.43, 95% CI = −3.35 to −1.51, *p* < 0.00001; heterogeneity: χ^2^ = 20.97, df = 7 (*p* = 0.0004); I^2^ = 67%]. Subgroup analysis based on the comorbidities was subsequently conducted, which showed a more meaningful result [Non-comorbidity: SMD = −1.43, 95% CI = −2.18 to −0.68, *p* = 0.0002; heterogeneity: χ^2^ = 3.95, df = 3 (*p* = 0.27); I^2^ = 24%; comorbidity: SMD = −3.37, 95% CI = −4.28 to −2.46, *p* < 0.00001; heterogeneity: χ^2^ = 3.46, df = 3 (*p* = 0.33); I^2^ = 13%]. Coincidentally, the two different subgroups belonged to two different MIRI models.

##### Anti-inflammation

Quantitative analysis of three studies (25, 37, 38) showed that luteolin administration significantly decreased TNF-α levels [Figure 4C, SMD = −2.88, 95% CI = −3.60 to −2.16, *p* < 0.0001; heterogeneity: χ^2^ = 1.83, df = 2 (*p* = 0.40); I^2^ = 0%].

## Discussion

### Summary of Results

This meta-analysis is the first to assess the effects of luteolin administration for treatment of MIRI. Comprehensive analysis was conducted of 18 studies with 415 animals. According to the checklist, the included studies were of relatively high quality with a low risk of bias. All the included data were objective response variables. The issues of comorbidities and different MIRI models were addressed in advance. Baseline data (i.e., administrations, dosages, pretreatment timing, and reperfusion duration) did not have a significant effect on effect size of IS and hemodynamics. Preclinical evidence obtained from the meta-analysis confirmed that pretreatment with luteolin reduced IS and ameliorated hemodynamics in animal models. These potential cardioprotective effects were primarily due to the anti-apoptosis, anti-oxidation, and anti-inflammation properties of luteolin.

### Limitations

The amount of data from mice is too small for further analysis. In regard to study quality, the included studies were credible but only four referred to a blind evaluation. Holman et al. (42) affirmed the necessity of blind outcome assessment while they also stated that experiment with an objective response variable did not need to be blind in animal researches. Hirst et al. (43) included 119,597 animals to evaluate the effects of blind outcome assessment which showed that subjective variable but not objective variable significantly increased effect sizes in the absence of blinding. In addition, there were notable differences among the experimental groups, which is likely the main reason for the absence of sample size calculation. As a result of the inclusion of more positive studies, the efficacy of luteolin may be overestimated. Besides, partial results were standardized by SMD with the 95% CI, thus the results should be carefully interpreted. The included studies employed multiple methods for the calculation of IS, which led to a smaller sample size in each subgroup.

### Implications

Recent basic research studies have reported the superior efficacy of flavonoids for the treatment of MIRI. Epidemiological investigations suggest that dietary intake of flavonoids could effectively reduce the risk of cardiac events (44, 45). As compared with other isolated flavonoids from Ixeris sonchifolia, luteolin was reported as the best option against ischemia-induced injury (46). Luteolin is mainly metabolized in the liver, and intestines and the metabolites are absorbed in the gastrointestinal tract (47). By oral administration, the highest plasma concentration of luteolin, which has a half-life of about 5 h, occurs about 1 h later (45). The anti-tumor properties of luteolin have been reported in wide range of studies, although the focus of most current research seems to be shifting to cardioprotective effects. The initial focus of this meta-analysis was not only MIRI, but all Post-infarction cardiac injuries. However, with the exception of one article (48), most studies screened the effect of luteolin for treatment of MIRI in accordance with stringent standards. However, the focus of the present meta-analysis was the effect of luteolin administration on MIRI.

#### Myocardial Infarction Size and Apoptosis of Cardiomyocytes

From the included studies, AAR had no statistical significance in any of the experimental groups. The goal of treatment is to reduce reperfusion injury to the viable myocardium stained by TTC. Apoptosis of cardiomyocytes was detected at the infarcted border zone in the animal models, while reperfusion of the infarcted area further induced apoptosis (49). In addition, clinical studies have reported a close association of apoptosis with MIRI (50). With the development and popularization of percutaneous coronary intervention, more studies have attached great importance to protecting cardiomyocytes at areas of greater risk. Luteolin was reported to reduce IS to varying degrees in different models and by different measurement methods.

The anti-apoptosis effect of luteolin as the end-stage outcome of the physiological impact is regulated by various mechanisms. Of these, the phosphoinositide 3-kinase (PI3K)/AKT pathway is considered to participate in cell survival (51). Yet, the high ratio of Bcl-2/Bax was negatively correlated with vulnerability to activation of apoptosis pathways (52, 53). The phosphorylation of AKT regulated by luteolin increased the ratio of Bcl-2/Bax and decreased the proportion of TUNEL-positive cells (22, 27). Luteolin also up-regulated the expression of the anti-apoptotic proteins FGFR2 and LIF, which was related to the activation of Akt signalling (29). The MIRI-induced decrease in sarcoplasmic reticulum Ca2+-ATPase (SERCA2a) activity was facilitated by luteolin partly through the PI3K/Akt signaling pathway. Luteolin activation of the PI3K/Akt pathway was reported to exert antioxidation effects in a simulated ischemia-reperfusion model (32), while the cardioprotective effects of luteolin were partly reversed by the PI3K inhibitors LY294002 and wortmannin (22, 26, 29).

C-Jun N terminal kinase (JNK), extracellular signal-regulated kinases (ERKs), and P38 are downstream effectors of the mitogen-activated protein kinase (MAPK) pathway (54). Members of the MAPK family regulate apoptosis of cardiomyocytes. Wu et al. (31) found that luteolin and the JNK-inhibitor SP600125 both attenuated cardiomyocyte apoptosis and that the JNK and ERK1/2 pathways have opposing relationships, as luteolin-mediated down-regulation of JNK and up-regulation of ERK1/2 had an anti-apoptotic effect in an MIRI model. Activated ERK1/2 mediated SERCA2a activity, while this positive effect was abolished by the ERK1/2 inhibitor PD98059 (31). During the reperfusion period, activated P38 MAPK resulted in Ca2+ overload and an imbalance in mitochondrial transmembrane potential. Then, the impaired mitochondria released pro-apoptosis proteins, which led to the loss of cardiomyocytes (40). Wei et al. (30) suggested that luteolin enhanced peroxiredoxin II activation to ameliorate mitochondrial dysfunction. Luteolin possesses an anti-apoptosis effect and improves SERCA2a activity, equal to that of the P38-inhibitor SB203580 (40). Yu et al. (35) demonstrated that luteolin via reactive oxygen species (ROS) activation of the MAPK pathways inhibited cardiomyocyte apoptosis in an MIRI model.

The anti-apoptosis effects of luteolin are also involved other mechanisms. For example, Bian et al. (20) suggested that luteolin exerted apoptotic protective effects through miR-208b-3p regulation of small interfering RNA Ets1 expression. SUMO1, a SUMO isoform, was found to convey cardioprotective effects in an MIRI model and luteolin improved SUMO1 expression to reduce cardiomyocyte apoptosis (21), while upregulating the expression of the transcription factor Sp1 to reduce cardiomyocyte death (23).

#### Oxidative Stress and Ca2+ Overload

During myocardial infarct, myocardiocyte metabolism switches to anaerobic respiration, which leads to the accumulation of lactate and Ca2+ overload, while the opening of mitochondrial membrane permeability transition pores (MPTPs) is Pre-vented by the acidic condition. During reperfusion, ROS generated from reactivation of the electron transport chain, xanthine oxidase, and nicotinamide adenine dinucleotide phosphate (NADPH) oxidase prompts the opening of MPTPs and causes sarcoplasmic reticulum dysfunction. With the elimination of lactic acid, restoration of the mitochondrial membrane potential results in Ca2+ overload in the mitochondria, which further induces the opening of the MPTPs (55). Reoxygenation of the heart inflicts significant myocardial injury throughout the ischemic region that exceeds the damage caused by ischemia alone (56). SERCA2a, an ATP-dependent enzyme, pumps Ca2+ into the sarcoplasmic reticulum to prevent Ca2+ overload.

Superoxide radicals combined with NO produce peroxynitrite, which results in myocardial dysfunction (57). Liao et al. (24) found that luteolin down-regulates the expression of inducible nitric oxide synthase (NOS), but had no effect on the expression levels of endothelial NOS (eNOS) and neuronal NOS. However, in a rat model of diabetes, eNOS was activated by luteolin to diminish oxidative stress induced by MIRI (32, 33). Xiao et al. (32) suggested that luteolin could activate eNOS to trigger antioxidative effects mediated by nuclear factor E2-associated factor 2 (Nrf2), which attenuated MIRI in diabetic rats. Moreover, the anti-oxidative effect was abolished by the NOS inhibitor L-NAME and the Nrf2 inhibitor brusatol. Nrf2, as the key molecule in redox balance, initiates the transcription of downstream antioxidant enzymes (58). The antioxidative function of Nrf2 was also improved by luteolin-mediated activation of the PI3K/Akt pathway. With a similar mechanism, Zhang et al. (36) revealed that luteolin attenuated oxidative injury through the PI3K/Akt pathway. ROS activation of members of the MAPK family was inhibited by luteolin to reduce MIRI (32, 36). In addition, luteolin enhanced the expression of the antioxidant protein peroxiredoxin II (30).

#### Inflammation and Hemodynamics

The release of ROS, cytokines, and complement results in the accumulation of neutrophils after the onset of myocardial reperfusion (55). The luteolin-mediated anti-inflammatory effect focuses on the inflammasome NLRP3/NF-KB pathway. Luteolin generally enhances the expression of various upstream binding factors, such as sirt1 and Toll-like receptor 4, to reduce inflammatory injury in MIRI (38). Hemodynamics can reflect the systolic and diastolic functions of heart. Effective cardiomyocyte contraction and relaxation can not be separated from calcium recycling. SERCA2a modulate cardiac cytosolic Ca^2+^ levels, SERCA Overexpression attenuates cardiac microvascular I/R injury through mitochondrial quality control (59, 60). The mitochondrial calcium uniporter (MCU) transports free Ca^2+^ into the mitochondrial matrix, maintaining Ca^2+^ homeostasis, thus regulates the mitochondrial morphology and energy supply (61). SERCA2a Overexpression inhibite the overactive MCU to reduce MIRI (62). Luteolin enhances SERCA2a activity via sumoylation of lysine 585 and Sp1 upregulation (21, 23) to improve hemodynamics in MIRI.

## Conclusions

The results of the present meta-analysis suggest that luteolin can act on different signaling pathways to reduce MIRI in animal models. As possible mechanisms, luteolin exerts anti-apoptosis, anti-oxidation, and anti-inflammation effects against MIRI. The main cardioprotective benefits of luteolin are the reduction of myocardial IS and the amelioration of intracardiac hemodynamics. There were some limitations to the methodology and study quality that reduce the strength of this evidence. Nonetheless, systematic inspection of these MIRI models provides preclinical evidence of the benefits of luteolin for clinical treatment of MIRI.

## Data Availability Statement

The original contributions presented in the study are included in the article/Supplementary Material, further inquiries can be directed to the corresponding authors.

## Author Contributions

QP and YL: contributed equally to this manuscript, wrote the manuscript, and made the figures. WM and RK: conducted the literature search, study selection, and analysis. DL and HZ: approved the final revisions of the manuscript submitted for publication. All authors contributed to the article and approved the submitted version.

## Funding

This work was supported by the Natural Science Foundation of China [grant number 81570326] and the Science and Technology Innovation Special Fund of Xuzhou Science and Technology Bureau [grant number KC17094].

## Conflict of Interest

The authors declare that the research was conducted in the absence of any commercial or financial relationships that could be construed as a potential conflict of interest.

## Publisher's Note

All claims expressed in this article are solely those of the authors and do not necessarily represent those of their affiliated organizations, or those of the publisher, the editors and the reviewers. Any product that may be evaluated in this article, or claim that may be made by its manufacturer, is not guaranteed or endorsed by the publisher.

## Supplementary Material

Supplementary Figure 1 Data merge formula.

Supplementary Figure 2 Egger test. **(A)** IS, **(B)** LVSP, (C) LVEDP, **(D)** +dp/dtmax, **(E)** -dp/dtmax, **(F)** apoptotic rate, **(G)** MDA, **(H)** TNF-α.

Supplementary Figure 3 3.1:trim and filling. **(A)** IS, **(B)** LVSP, **(C)** LVEDP, **(D)** +dp/dtmax, **(E)** -dp/dtmax, **(F)** apoptotic rate, **(G)** MDA, **(H)** TNF-α. 3.2: funnel plot **(a)** IS, **(b)** LVSP, **(c)** LVEDP, **(d)** +dp/dtmax, **(e)** -dp/dtmax, **(f)** apoptotic rate, **(g)** MDA, **(h)** TNF-α.

Supplementary Figure 4 Pooled estimates of myocardial infarct size for luteolin vs. vehicle in different MIRI models. Model of LAD ligation: the model was constructed by the left anterior descending coronary artery ligation. Model of global ischemia: heart was subjected to global no-flow ischemia without pacing.

Supplementary Figure 5 Sensitivity analysis.

Supplementary Figure 6 Sensitivity analysis.

Supplementary Figure 7 Sensitivity analysis.

Supplementary Table 1 Regression analysis in the model of LAD ligation and global ischemia.
