# Supplementary figures and images for: Cardioprotective Effects and Possible Mechanisms of Luteolin for Myocardial Ischemia-Reperfusion Injury: A Systematic Review and Meta-Analysis of Preclinical Evidence
Source: Front Cardiovasc Med. 2022 Apr 25;9:685998. doi: 10.3389/fcvm.2022.685998 (PMC9081501; doi:10.3389/fcvm.2022.685998)

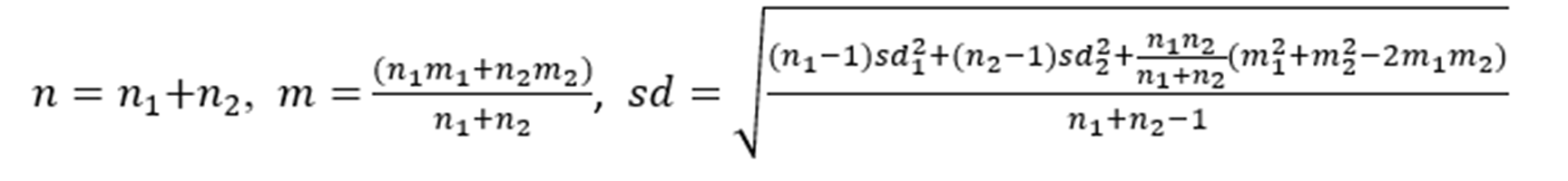

Supplement: Supplementary Figure 1 — Data merge formula. [file Image_1.TIF]

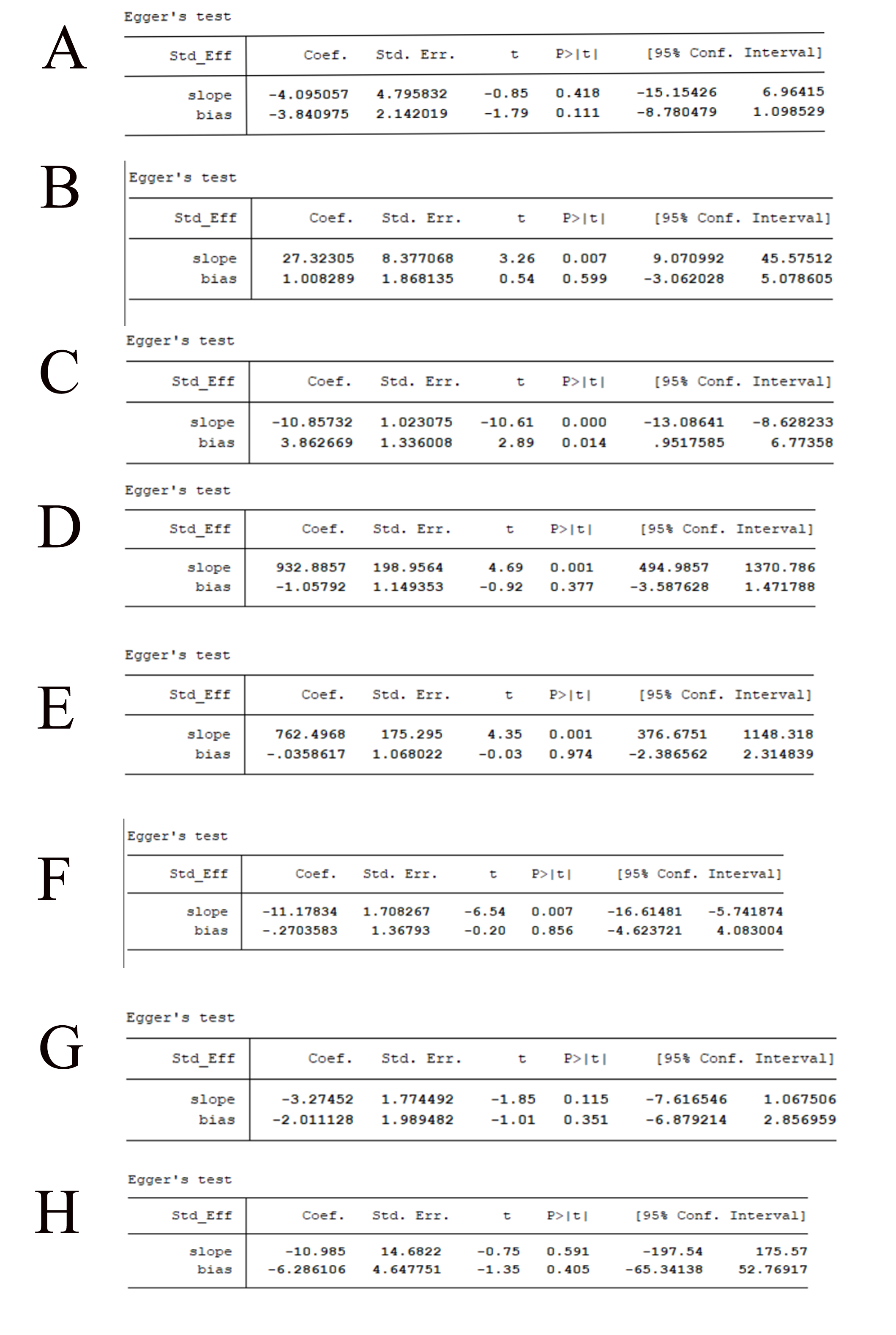

Supplement: Supplementary Figure 2 — Egger test. (A) IS, (B) LVSP, (C) LVEDP, (D) +dp/dtmax, (E) -dp/dtmax, (F) apoptotic rate, (G) MDA, (H) TNF-α. [file Image_2.TIF]

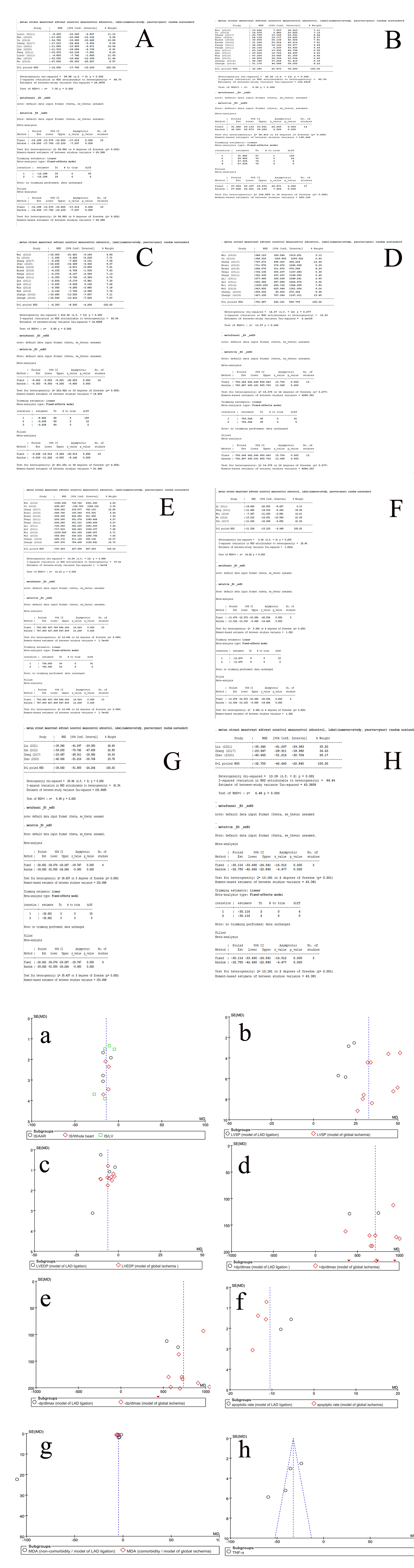

Supplement: Supplementary Figure 3 — 3.1:trim and filling. (A) IS, (B) LVSP, (C) LVEDP, (D) +dp/dtmax, (E) -dp/dtmax, (F) apoptotic rate, (G) MDA, (H) TNF-α. 3.2: funnel plot (a) IS, (b) LVSP, (c) LVEDP, (d) +dp/dtmax, (e) -dp/dtmax, (f) apoptotic rate, (g) MDA, (h) TNF-α. [file Image_3.TIF]

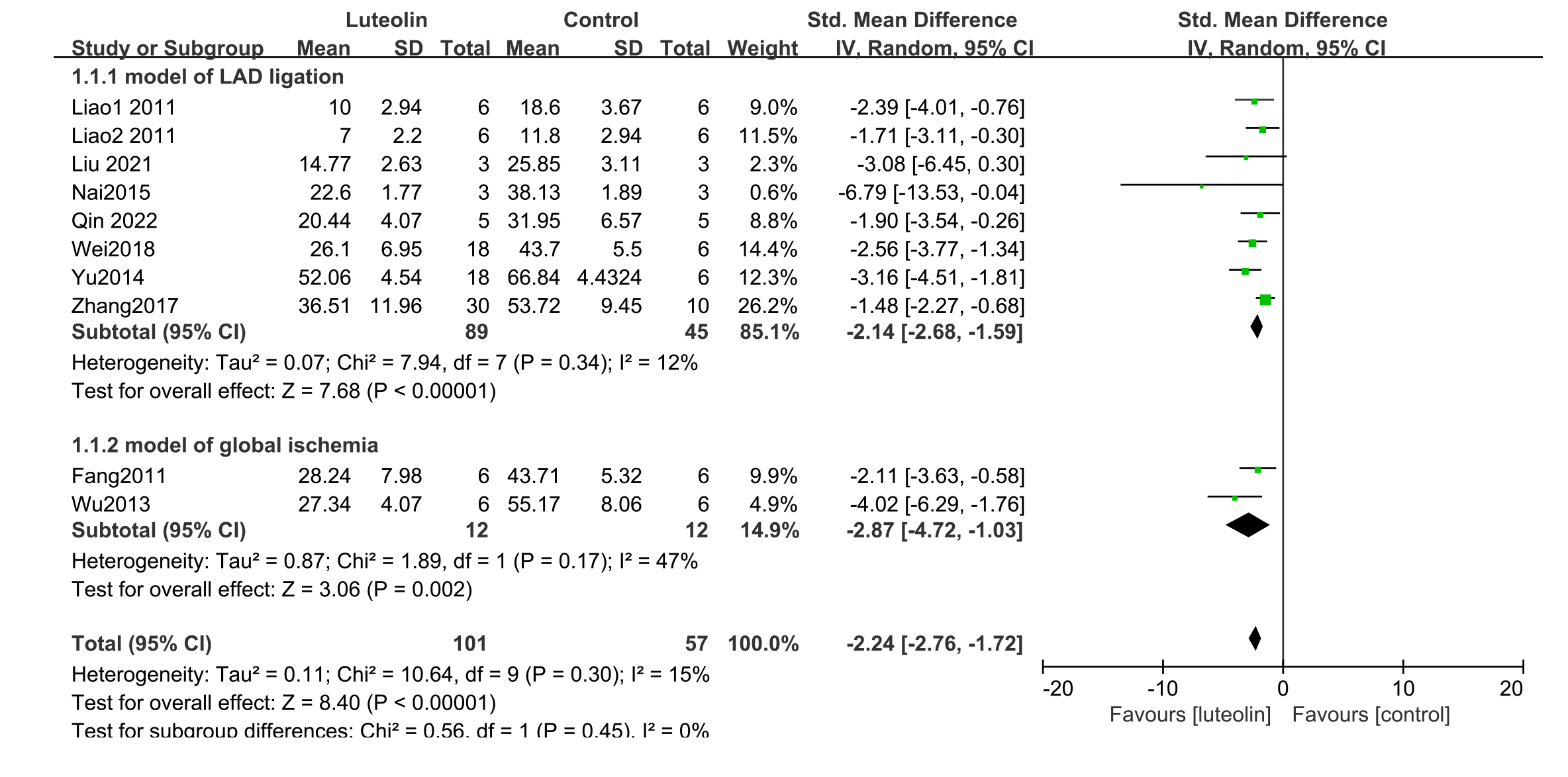

Supplement: Supplementary Figure 4 — Pooled estimates of myocardial infarct size for luteolin vs. vehicle in different MIRI models. Model of LAD ligation: the model was constructed by the left anterior descending coronary artery ligation. Model of global ischemia: heart was subjected to global no-flow ischemia without pacing. [file Image_4.TIF]

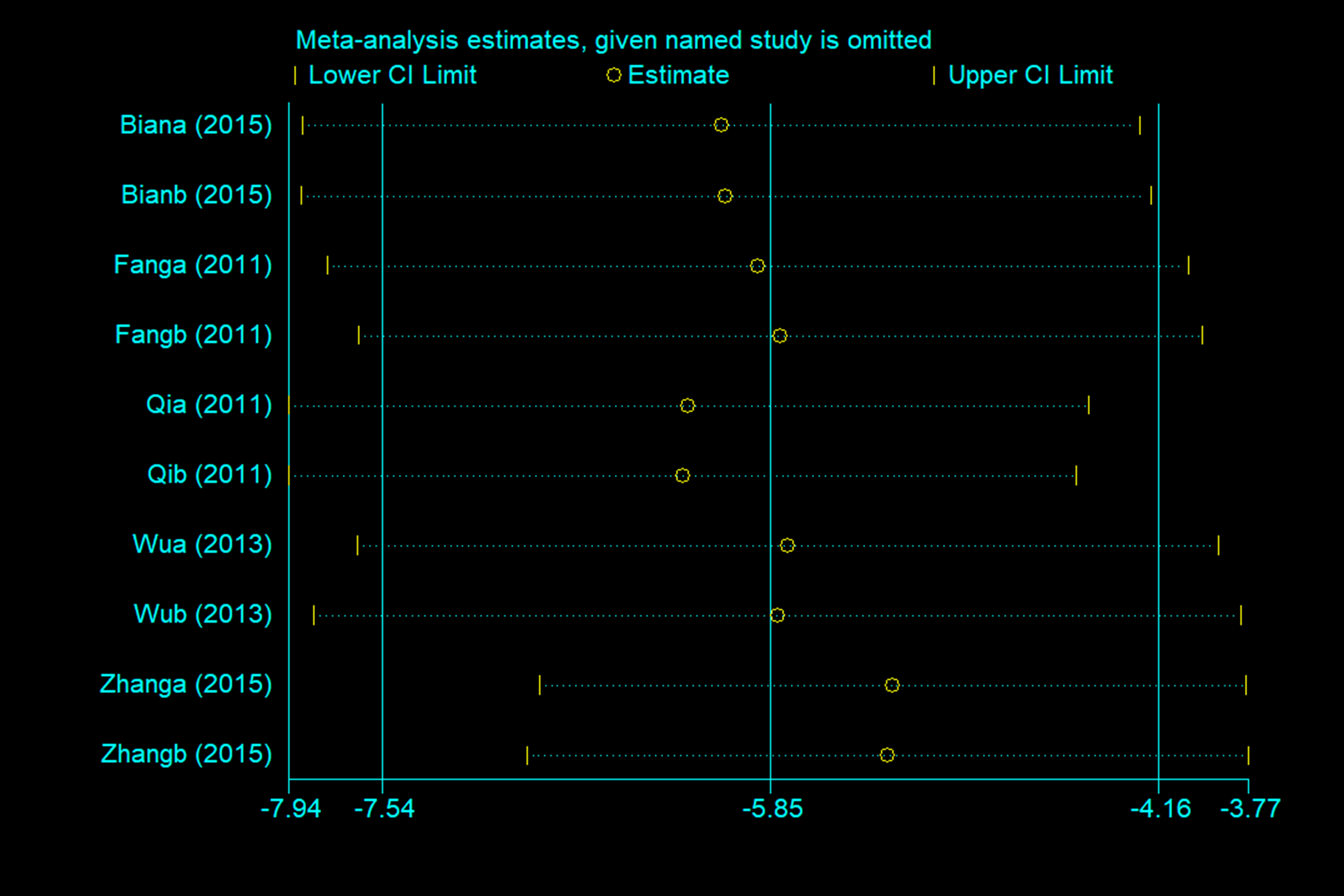

Supplement: Supplementary Figure 5 — Sensitivity analysis. [file Image_5.TIF]

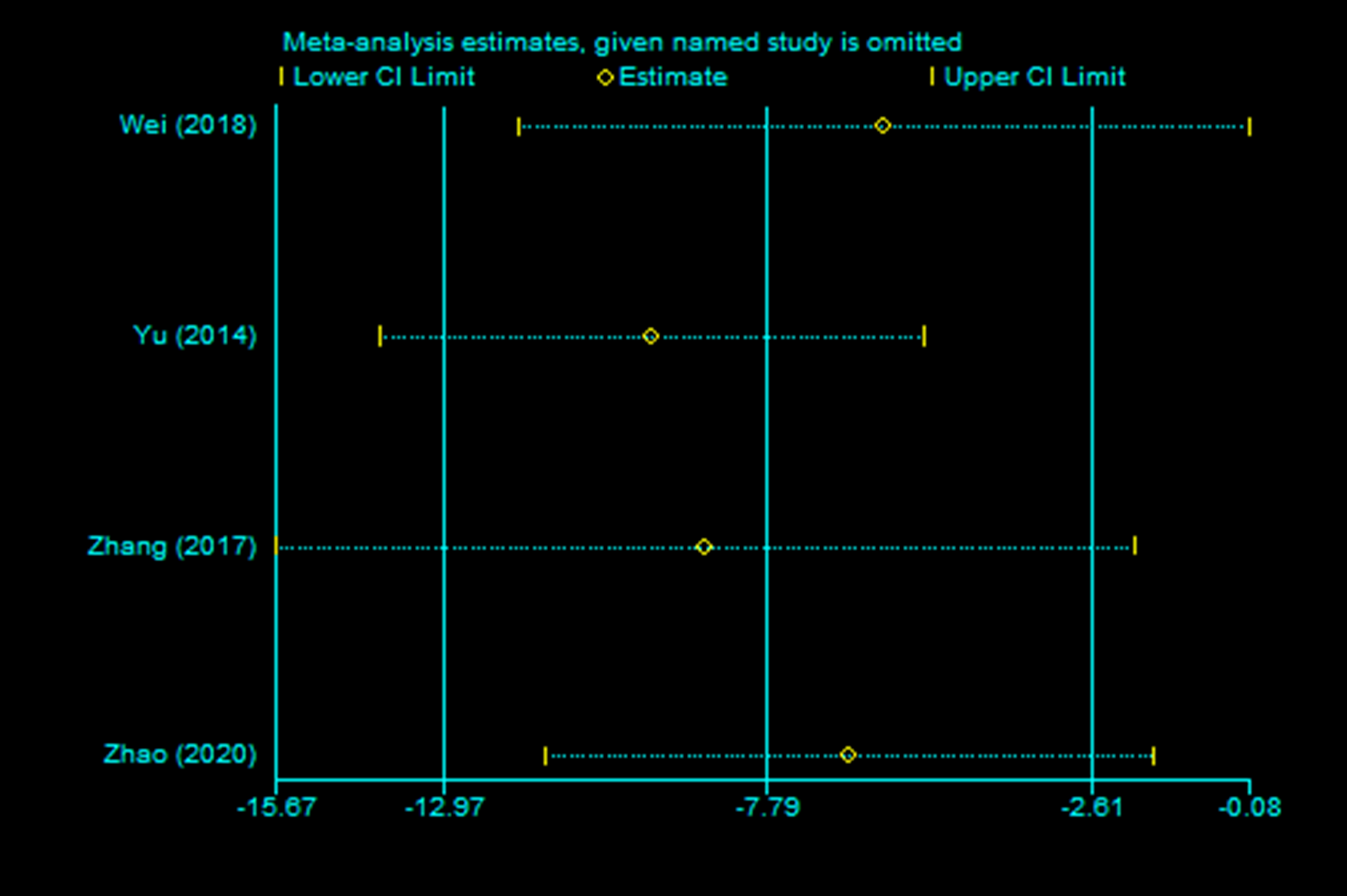

Supplement: Supplementary Figure 6 — Sensitivity analysis. [file Image_6.TIF]

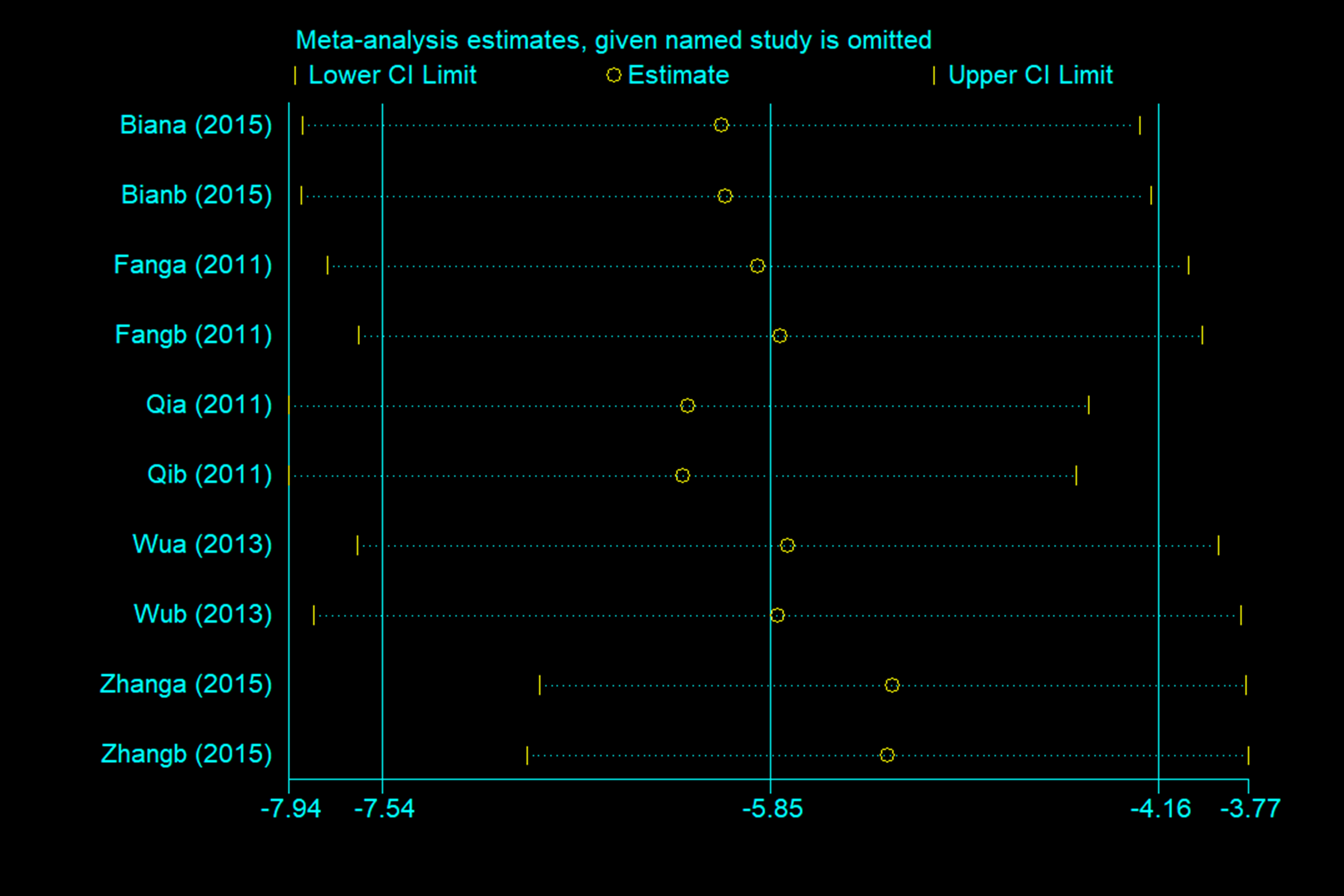

Supplement: Supplementary Figure 7 — Sensitivity analysis. [file Image_7.TIF]
