# Supplementary material for: Cardioprotective Effects and Possible Mechanisms of Luteolin for Myocardial Ischemia-Reperfusion Injury: A Systematic Review and Meta-Analysis of Preclinical Evidence
Source: Front Cardiovasc Med. 2022 Apr 25;9:685998. doi: 10.3389/fcvm.2022.685998 (PMC9081501; doi:10.3389/fcvm.2022.685998)
Supplement: Supplementary Table 1 — Regression analysis in the model of LAD ligation and global ischemia. [file Table_1.docx]

**Supplement Table 1** regression analysis in the model of LAD ligation and global ischemia

|  | *P* value | | | | |
| --- | --- | --- | --- | --- | --- |
|  | model of LAD ligation | | | | model of LAD ligation |
|  | timing regimen of pretreatment | dosages | reperfusion duration | administration | reperfusion duration |
| IS | 0.215 | 0.436 | 0.162 | 0.601 | - |
| LVSP | 0.506 | 0.902 | 0.768 | 0.385 | 0.793 |
| LVEDP | 0.617 | 0.360 | 0.428 | 0.095 | 0.926 |
| **+**dp/dt_max_ | 0.710 | 0.866 | 0.479 | insufficient observations | 0.389 |
| -dp/dt_max_ | 0.291 | 0.462 | 0.653 | insufficient observations | 0.497 |
